# Activation of Autophagy Through the NLRP3/mTOR Pathway: A Potential Mechanism for Alleviation of Pneumonia by QingFei Yin

**DOI:** 10.3389/fphar.2021.763160

**Published:** 2022-01-17

**Authors:** Xiaozhou Sun, Dandan Wang, Lizhong Ding, Yan Xu, Wenxiu Qi, Daqing Zhao, Li Liu, Chengcheng Yin, Changsheng Cui, Zhongtian Wang, Liwei Sun, Liping Sun

**Affiliations:** ^1^ College of Chinese Medicine, Changchun University of Chinese Medicine, Changchun, China; ^2^ Research Center of Traditional Chinese Medicine, The Affiliated Hospital to Changchun University of Chinese Medicine, Changchun, China; ^3^ Center of Children’s Clinic, The Affiliated Hospital to Changchun University of Chinese Medicine, Changchun, China; ^4^ Jilin Provincial Key Laboratory of Bio Macromolecules of Chinese Medicine, Jilin Ginseng Academy, Changchun University of Chinese Medicine, Changchun, China; ^5^ College of Pharmacy, Changchun University of Chinese Medicine, Changchun, China

**Keywords:** proteomics, *Streptococcus pneumoniae* pneumonia, NLRP3, autophagy, QingFei Yin

## Abstract

QingFei Yin (QFY), a Chinese traditional medicine recipe, is known for its excellent therapeutic pharmacological effects for the treatment of bacterial lung infections, although its molecular mechanism of action remains unknown. Here, QFY chemical composition was determined using a High-Performance Liquid Chromatography-Mass (HPLC-MS/MS)-based method then QFY was evaluated for protective pharmacological effects against pneumonia using two models: a *Streptococcus pneumoniae*-induced *in vivo* mouse model and an *in vitro* pneumolysin (PLY)-induced murine lung alveolar-derived MH-S cell line-based model. Notably, QFY exerted prominent anti-pneumonia effects both *in vivo* and *in vitro*. To further explore QFY protective effects, 4D label-free proteomics analysis, pathologic evaluation, and immunohistochemical (IHC) analysis were conducted to identify cellular pathways involved in QFY protection. Notably, our results indicated that NF-κB/NLRP3 and autophagy pathways may contribute to pharmacological effects associated with QFY-based protection. Briefly, QFY triggered autophagy *via* down-regulation of upstream NLRP3/mTOR signaling pathway events, resulting in the amelioration of inflammatory injury. Collectively, our results revealed molecular mechanisms underlying QFY protection against pneumonia as a foundation for the future development of novel treatments to combat this disease and reduce antibiotic abuse.

## Introduction

Pneumonia, a type of acute lower respiratory infection, accounts for an extremely large proportion of the overall worldwide disease burden (Rudan et al., 2008; D 2016 Lower Respirator, 2018; Bernstein, 1999). *Streptococcus pneumoniae* (*S.pn*), a gram-positive bacterium, is recognized as the most common cause of pathogen-induced pneumonia, while also causing respiratory tract infections, pulmonary parenchymal inflammation, and pulmonary infection-derived bacteremia (D 2016 Lower Respirator, 2018; Bernstein, 1999). Such dire pathological effects have been shown to involve the pneumococcal cytolysin pneumolysin (PLY), a major virulence factor expressed by almost all pneumonia-causing *S.pn* bacteria (Bewley et al., 2014; Subramanian et al., 2019). Importantly, PLY toxin possesses a unique feature whereby it can induce host pore-dependent pro-inflammatory responses (Subramanian et al., 2019) that appear to play major roles in pneumococcal pathogenicity.

The advent of antibiotic use has greatly diminished pneumonia mortality rates. However, the emergence and spread of antibiotic resistance worldwide has directly resulted in the need for physicians to prolong administration of pneumonia treatments, leading to increased incidence of complications and greater mortality (Bakkeren et al., 2020; Lehtinen et al., 2020; Lewnard et al., 2020). Therefore, treatment strategies for pneumonia must be administered with extraordinary care by the biomedical community (Quinton et al., 2018). Meanwhile, traditional Chinese medicines (TCM) as alternative treatments have been attracting increasing attention around the world, due to their long history of clinical use and proven therapeutic pharmacological effects for alleviating infectious diseases (Qiu et al., 2012; Chen et al., 2015; Yang et al., 2017; Gong et al., 2020; Zhang et al., 2020). Qing Fei Yin (QFY) is one such Chinese traditional medicine recipe that contains *Scutellaria baicalensis* Georgi [Lamiaceae; Scutellariae radix], *Forsythia suspensa* (Thunb.) [Oleacea; Forsythiae Fructus], *Belamcanda chinensis* (L.) DC [Iridaceae; Elamcandae rhizoma], and *Fritillaria cirrhosa* D. Don [Liliaceae; Fritillaria Cirrhosae bulbus]. QFY evolved from the “Gan Lu Xiao Du pill” that was described in the classic Chinese book “Yi Xiao Mi Chuan,” a reference guide that is widely used to guide the clinical treatment of pneumonia. The excellent therapeutic effect of QFY against pneumonia highlights its potential as a therapy that will likely help clinicians avoid using antibiotics to treat this disease. Nevertheless, the molecular mechanism underlying QFY alleviation of pneumonia remains unclear.

High-throughput proteomics tools have been widely recognized as useful for exploring the pharmacology of complex TCM systems (Zhang et al., 2014; Dai et al., 2018; Shen et al., 2019). Therefore, in this study the *in vivo* curative effect of QFY was evaluated in *S.pn*-infected mice, with changes in protein expression before and after QFY treatment systematically compared using a method based on application of 4D label-free proteomics analysis. Our results identified 655 and 345 differentially expressed proteins based on comparisons of expression levels between the Control group versus *S.pn* group and between the *S.pn* + QFY group versus *S.pn* group, respectively. Subsequently, GO (gene ontology) and KEGG (Kyoto Encyclopedia of Genes and Genomes) pathway analysis strongly implicated involvement of more than 40 differentially expressed proteins associated with NOD-like receptor-dependent and autophagy pathway-dependent functions. These results prompted us to surmise that NLRP3 and autophagy were both associated with development of *S.pn*-induced infectious disease and highlight their potential value as targets of QFY-based therapy.

It has become evident that the host inflammatory reaction to a pathogen can play a predominant role in infectious disease outcomes. Recent studies have suggested that macrophage surface and intracellular toll-like receptors (TLRs) and nucleotide-binding oligomerization domain (NOD)-like receptors (NLRs) may participate in pathogen sensing and inflammatory responses (Klein et al., 2008; Swanson et al., 2019). Unlike TLRs, which directly recognize their agonists, NLRs are associated with inflammasome activation and caspase-1 (cysteinyl aspartate-specific proteinase-1)-dependent pro-IL-1β/IL-18 release. NLRP3 (NOD-like receptor protein 3), a well-known member of the NLRs family, binds to its adaptor protein ASC (apoptosis-associated speck-like protein containing a CARD, where CARD signifies a C-terminal caspase recruitment domain). Activation of the NLRP3 inflammasome requires two signals, including an initial priming signal regulated by NF-κB and an assembly signal that triggers caspase-1 cleavage (Klein et al., 2008; Fang et al., 2011; Harris et al., 2017). Meanwhile, mounting evidence has revealed a bidirectional modulatory effect of NLRP3 on bacterial invasive disease. More specifically, during the initial period of *S.pn* invasion, NLRP3 contributes to protective immunity and host defense (Dela Cruz et al., 2012; Lemon et al., 2015; Hassane et al., 2017). However, during late-stage infection, PLY stimulation leads to increased NLRP3 expression that induces alveolar epithelial cells and macrophages to over-release mature inflammatory cytokines. Increased levels of these cytokines subsequently enhance the inflammatory response and ultimately promote progression to pneumonia (McNeela et al., 2010a; Hoegen et al., 2011; Bewley et al., 2014; van Lieshout et al., 2018).

Aside from NOD-like receptor-based host defenses, autophagy has recently attracted attention as a critically important homeostatic process involved in host defenses and multicellular-based immunity. Importantly, autophagy acts to reduce bacterial burden and microbial tissue damage by resisting invading pathogens (Hu et al., 2016). Consequently, promotion of autophagy to degrade NLRP3 may be an effective therapeutic strategy for preventing and treating *S.pn*-induced pneumonia. Nevertheless, it is important to note that NLRP3 and autophagy are linked by reciprocal regulatory processes (Shi et al., 2012; Cadwell, 2016; Cosin-Roger et al., 2017). For example, it appears that degradation of NLRP3 partly depends on autophagy (Nakahira et al., 2011). Indeed, accumulating evidence suggests that such processes may function within a bidirectional regulatory network linking NLRP3 to autophagy (Zhou et al., 2011). Meanwhile, NLRP3 has been identified as a binding partner of mTOR, whereby NLRP3 mechanistically inhibits autophagy by promoting mTOR phosphorylation. In turn, autophagy is an essential process for governing NLRP3 degradation (Cosin-Roger et al., 2017). Therefore, NLRP3 and autophagy are potential treatment targets for use in combating *S.pn*-based infectious diseases. However, complex bidirectional regulatory mechanisms operating within the NLRP3-autophagy axis and the precise target of QFY action remain unclear and were therefore investigated comprehensively in this work.

## Materials and Methods

### Materials and Reagents

*S. pneumoniae* serotype 2 strains D39, a type 2 pneumococcal strain, was provided by Zunyi Medical College. Culture medium components were purchased from commercial vendors as follows: PLY from Fitzgerald (Acton, MA, United States), Todd Hewitt broth from Difco (Detroit, MI, United States), RPMI 1640 medium from Gibco (New York, NY, United States), and fetal bovine serum (FBS) from CLARK Bioscience (Claymont, DE, United States). The MH-S cell line was purchased from the Cell Bank of the Chinese Academy of Sciences and was derived from alveolar macrophages of 7-week-old mice (Shang Hai, China). Mouse interleukin-lβ (IL-1β, #MLB00C), tumor necrosis factor-alpha (TNF-α, #MTA00B), and interleukin-6 (IL-6, #M6000B) enzyme-linked immunosorbent assay (ELISA) kits were obtained commercially (R&D Systems, Minneapolis, MN, United States). Rabbit polyclonal antibodies against ULK (#ab167139), p62 (#ab155686), NF-κB P65 (#ab16502), NF-κB p-P65 (#ab194726), IκBα (#ab7217) LC3B (#ab51520), and β-tubulin (#ab18207) were purchased from Abcam (Cambridge, MA, United States). Rabbit monoclonal antibodies against mTOR (#ab32028), p-mTOR (#ab109268), and p-ULK (#ab229909) and mouse monoclonal antibodies against GAPDH (#ab8245) were purchased from Abcam. Rabbit polyclonal antibodies against NLRP3 (#15101S), ASC (#67824T), and cleaved casp1 (#89332S) were purchased from Cell Signaling Technology (Beverly, MA, United States). 3-Methyladenine, a PI3K inhibitor and inhibitor of autophagy (3-MA, #HY-19312) was purchased from MCE (Beverly, MA, United States).

### Preparation of the QFY Aqueous Extract

QFY was provided by the Affiliated Hospital of Changchun University of Traditional Chinese Medicine (Jilin, China). Each component powder was accurately weighed and formulated to generate QFY based on the following ratio (by weight): *Scutellaria baicalensis* Georgi*, Forsythia suspensa* (Thunb.), *Belamcanda chinensis* (L.) DC, and *Fritillaria cirrhosa* D. Don of 8:8:8:3. According to the standard procedure (National Pharmacopoeia Committee, 2005), QFY powder was decocted three times with 200 ml of water at 100°C to obtain the aqueous extract. All decocted solutions were combined and centrifuged then the supernatant was dried under vacuum to produce a brown powder. The extract was stored in the laboratory at −80°C until use.

### Components Identification of QFY

In order to identify compounds within the QFY extract, the extract was subjected to HPLC-MS/MS conditions using a chromatography system (Thermo, Ultimate 3000LC, Q Exactive HF), with separations conducted using a Zorbax Eclipse C18 column (1.8 μm, 2.1 × 100 mm). The mobile phase consisted of 0.1% formic acid water (phase A) and acetonitrile (phase B) with a flow rate of 0.3 ml/min, a 1:1 splitter ratio and gradient elution. Gradient change parameters for acetonitrile (B) were 0–2 min, 5% B; 2–6 min, 30% B; 6–7 min, 30% B; 7–12 min, 78% B; 12–14 min, 78% B; 14–17 min, 95% B; 17–20 min, 95% B; 20–21 min, 5% B; 21–25 min, 5% B. 2 μl of sample was injected at room temperature. The mass spectrometer was operated in both positive and negative ion modes. The following instrument parameters were applied: heater temperature of 325°C, ion spray voltage of 3.5 kV, sheath gas flow velocity of 45 arb, auxiliary gas velocity of 15 arb, scavenging air velocity of 1 arb, and S-Lens RF Level of 55%. Scanning modes included full scan (*m/z* 100–1500) and data-dependent second-order mass spectrometry scanning (dd-MS2, TopN = 10). The collision mode was set to high energy collision dissociation (HCD). Compound Discoverer 3.1 was used for retention time correction, peak identification, peak extraction, etc. Compounds were identified based on comparisons to secondary mass spectrometry spectra of compounds within the thermo mzcloud online database and thermo mzvalut local database. HPLC was conducted as described previously (Wang et al., 2012; Li et al., 2016; Huang et al., 2018). Details regarding experimental conditions are presented in the Supplementary Material section (Methods and materials, section 1.3).

### Bacterial Strains and Culture Conditions

The *S.pn* strain D39 (Berry et al., 1989) was cultured at 37°C in Todd Hewitt broth then the culture was inoculated onto tryptic soy broth (TSB) agar plates and incubated under the same conditions overnight. Next, the bacterial fluid was collected from the plates and cultured in Todd Hewitt broth supplemented with 5% yeast extract until growth reached mid-log phase (as determined by optical density at 600 nm).

### Animals

Female BALB/c mice (the Animal Ethics Committee of Changchun University of Chinese Medicine-20190116), which had been housed and maintained for 6 weeks until they weighed 20 ± 2 g, were purchased from the Experimental Animal Center of Changchun University of Traditional Chinese Medicine. The mice were allowed to rest for 7 days to acclimate before being subjected to experimental manipulations; with all procedures complying with guidelines set forth by the ACUC (Animal Care and Use Committee) affiliated with the Changchun University of Traditional Chinese Medicine.

First, 24 mice were randomly divided into four groups: Control group, *S.pn* group, *S.pn +* QFY (L) group (0.21 g/kg, aqueous extract), and *S.pn +* QFY (H) group (0.42 g/kg, aqueous extract), where (L) denotes low-dose and (H) denotes high-dose QFY. Pneumonia was induced according to previously reported experimental methods (Dessing et al., 2010). Briefly, mice in *S.pn*, *S.pn +* QFY(L), and *S.pn +* QFY(H) groups were lightly anesthetized *via* inhalation of isoflurane then were infected *via* left nasal inoculation of a 25-μl volume of nose drops containing 2.5 × 10^8^ CFU/ml of *S.pn* to establish the *in vivo* pneumonia model. Meanwhile, beginning at the time of *S.pn* infection, mice in *S.pn* + QFY (L) and *S.pn* + QFY (H) groups were administrated QFY by means of intragastric administration twice daily. In these experiments, all mice were sacrificed at 48 h post-infection.

### Hematoxylin-Eosin (HE) Staining of Lung Tissues

Lung tissues of mice were prepared for histology and analyzed as previously described (Dessing et al., 2010) by immersing tissue specimens in 4% paraformaldehyde followed by paraffin embedding, sectioning, and dewaxing of slices with xylene, with ethanol washes performed between steps. After water was removed *via* dehydration, slices were stained with hematoxylin for 5 min. After rinsing slices in hydrochloric acid mixed with ethanol (differentiation step) for 30 s, slices were soaked in distilled water for 15 min, immersed in eosin staining solution for 2 min, then were dewaxed until transparent and sealed.

To score lung inflammation and damage, the entire lung surface was analyzed with respect to the following parameters: interstitial damage, vasculitis, peri-bronchitis, edema, thrombus formation, and pleuritis. Each parameter was graded on a scale of 0–4, (0: absent; 1: mild; 2: moderate; 3: severe; 4: very severe) (Nouri-Aria et al., 2004; Dessing et al., 2007). Percentages of lung surfaces exhibiting signs of pneumonia were scored and graded according to the following scale: 0–4 (0: absent; 1: 5–20% confluent pneumonia; 2: 21–40%; 3: 41–60%; 4: 61–80%; 5: 81–100%). The total lung inflammation score was expressed as the sum of the scores for each parameter, with a maximum attainable score of 24 (Renckens et al., 2007).

### Bronchoalveolar Lavage

Briefly, the trachea was exposed through a midline incision and bronchoalveolar lavage fluid (BALF) was harvested by instilling and retrieving two 0.5-ml aliquots of sterile isotonic saline (Dessing et al., 2007). BALF was centrifuged at 3000 rpm for 5 min, frozen at −80°C, then was analyzed *via* ELISA for inflammatory factors such as IL-6, IL-1β, and TNF-α.

### Quantitative Proteomics Analysis

Proteomics analysis was conducted by Jingjie PTM BioLabs (Hangzhou, China). Primary experimental procedures for 4D Label free proteomics analysis included protein preparation, trypsin digestion, HPLC fractionation, LC-MS/MS analysis, and bioinformatics analysis.

#### Protein Extraction and Digestion

Proteins were extracted from lung tissues of mice in Control, *S.pn*, and *S.pn* + QFY groups as previously described (Jianye et al., 2018). Briefly, after each sample was frozen in liquid nitrogen, it was ground into a powder to which lysis buffer (8 M urea, 1% Protease Inhibitor Cocktail) was added followed by three rounds of sonication on ice using a high-intensity ultrasonic processor (Scientz, China). Remaining debris were removed by centrifugation 12,000 r/min at 4°C for 10 min. Finally, the supernatant was collected, and the protein concentration was determined using a BCA kit according to the manufacturer’s instructions.

#### Quantitative Proteomic Analysis *via* LC-MS/MS and Data Analysis

The tryptic peptides were dissolved in solvent A (0.1% FA, 2% ACN in water) then were separated on using a home-made analytical column (25-cm length, 100-μm i.d.) using a nanoElute UHPLC System (Bruker Daltonics) with solvent B (0.1% FA in ACN) gradient increases conducted as follows: from 4 to 22% over 70 min, 22–30% over 14 min, 80% over 3 min, with a 3-min equilibration period between gradient changes and a flow rate of 450 nl/min. Peptides were subjected to capillary HPLC to concentrate them followed by mass spectrometry using a tims TOF Pro (Bruker Daltonics) system. Precursors with charge states of 0–5 and fragments detected within the MS/MS scan range of 100–1700 m/z were analyzed using the TOF detector; with 10 PASEF-MS/MS scans acquired per cycle.

The resulting MS/MS data were processed using MaxQuant (v.1.6.6.0) with an integrated Andromeda search engine. Tandem mass spectra were searched against the Swissprot Mouse database (17,032 entries) then were concatenated using the reverse decoy database. The false discovery rate for proteins and peptides was adjusted to 1%.

#### Bioinformatics Analysis

In the present study, ontology-based pathway analysis was conducted using the Gene Ontology (GO) Consortium-based vocabulary of terms. Subcellular localization prediction, functional enrichment, and cluster analysis were all performed using GO terms and KEGG pathways. Cluster membership was visualized using a heat map developed using the “heatmap.2” function from the “plots” R-package. Fold changes in protein levels for Control, *S.pn*, and *S.pn* + QFY group comparisons were calculated as mean values according to relative and absolute quantification ratios of protein isobaric tags. Student’s t-tests were conducted using SPSS 25.0 to assess statistical significance of results, with proteins exhibiting significant differential expression (*p* < 0.05) with an expression fold-change >2 identified as differentially expressed biomarkers.

### Western Blot Analysis

Western blot analysis of proteins obtained from lysates of cells or lung tissue homogenates were performed as described previously (Zahlten et al., 2013). 30 µg of each lysate was separated/lane using 10% SDS-PAGE gels. Primary antibodies specific for the following antigens were used at 1:1000 dilution: β-tubulin, GAPDH, NLRP3, ASC, cleaved-casp1, LC3B, P62 p-mTOR, mTOR, p-ULK, ULK, NF-κB p65, p-NF-κB p65, and IκB. After blots were incubated with primary antibodies overnight at 4°C, they were incubated with secondary horseradish peroxidase-conjugated IgG (either goat anti-mouse or goat anti-rabbit) diluted 1:5000. Protein bands were visualized and analyzed using a chemiluminescent imaging system (FluorChem, ProteinSimple, San Jose, CA, United States).

### Immunohistochemistry

Immunohistochemistry was performed on mouse lung tissues after they were paraffin-embedded, sectioned, and deparaffinized, hydrated, and heated in a low-pH citrate buffer using a microwave oven to render intracellular antigens accessible to immunostaining. Subsequently, sections were separately immunostained with rabbit polyclonal antibodies overnight at 4°C. Next, sections were submerged in a hydrogen peroxide solution for 5 min at room temperature to block endogenous peroxidase activity followed by treatment with corresponding fluorescent antibody for 60 min at room temperature. Fluorescently stained sections were then counterstained with DAPI solution (nuclear staining) for 5 min then were dried and sealed with a reagent to prevent fluorescence quenching during storage. Sections were observed under a Nikon inverted fluorescence microscope (Nikon Eclipse Ti-SR, Nikon, Japan) and images were captured with a DS-U3 system (Nikon).

### Cell Culture

MH-S cells of 5th passage were cultured at 37°C in 5% CO_2_ in 1640 medium supplemented with 10% FBS and 1% penicillin-streptomycin. MH-S cells were stimulated with purified PLY (400 ng/ml) to generate the *in vitro* lung injury model. Meanwhile, beginning at the time of PLY infection, cells were treated with QFY (50 μg/ml) for 24 h followed by treatment with or without 3-MA (10 ng/ml) for 12 h before cells were collected for further analysis (Zhao et al., 2016).

### RNA Interference

MH-S cells were seeded into wells of six-well plates. Next, the cells were transfected with NLRP3 small-interfering RNA and control siRNA (Sangon Biotech, Shanghai) using LipofectAmine 2000 reagent (Invitrogen) in serum-free RPMI 1640 medium as per the manufacturer’s instructions. At 24 h after transfection, cells were incubated with PLY and QFY as described above.

### Statistics

All data are presented as means ± SD as calculated using Student’s *t*-test Comparisons between groups were conducted using one-way or two-way ANOVA followed by Bonferroni indicated. Differences were considered significant at Control group vs *S.pn* group or PLY group, *p < 0.05, **p < 0.01, *S.pn* group or PLY group vs *S.pn* + QFY groups or PLY + QFY groups, ^#^ *p* < 0.05, ^##^ *p* < 0.01, with n.s. used to indicate results were not significant.

## Results

### HPLC Chromatograms of QFY Formulations

**FIGURE 1:**
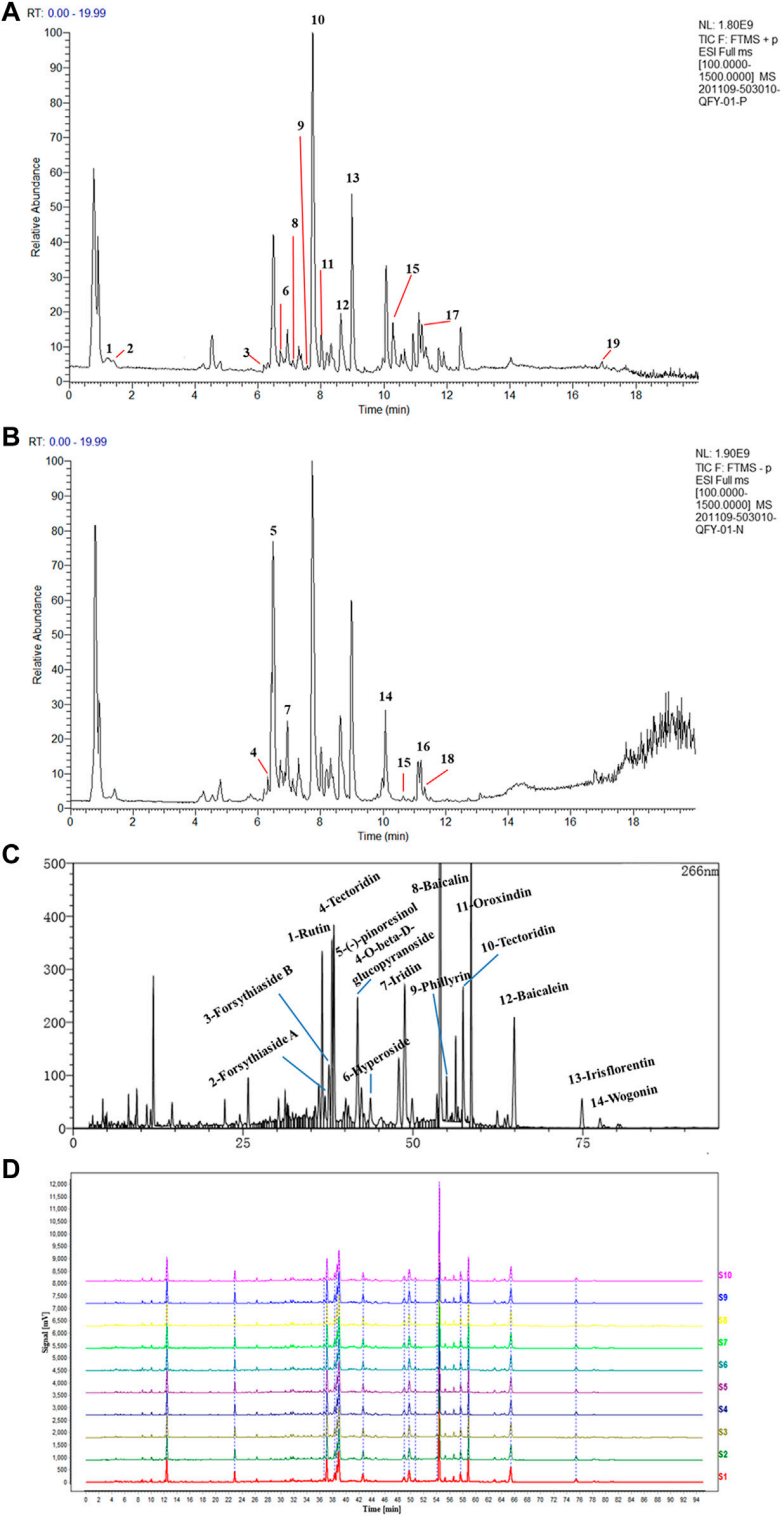
HPLC chromatograms of QFY formulations. Total Ion Current (TIC) of QFY formulation extracts in both positive **(A)** and negative **(B)** ion models. The peaks of 1–19 are listed in Table 1. **(C)** HPLC chromatogram of QFY formulation. **(D)** HPLC fingerprint chromatograms of 10 batches of QFY (S1–10) formulations analyzed using National Pharmacopeia Committee Chinese Medicine Fingerprint Similarity Evaluation System (2004A) software, with UV detection at 266 nm.

HPLC-MS/MS methods have been widely used to analyze major compounds in various herbal medicines. In this study, 19 main compounds of QFY were identified using this method, with total ion current (TIC) values for these 19 components shown in Figures 1A,B. To achieve precise identification of QFY components, Compound Discoverer 3.1 was used for retention time correction, peak identification, and extraction. Information regarding compound name, molecular formula, retention time (RT), mass accuracy, and adduct is presented in Table 1. Consistent with our HPLC-MS/MS results, 14 main peaks within the HPLC fingerprint were attributed to QFY compounds (Figure 1C). Due to the fact that proportions of cytidine, adenosine, caffeic acid, phillyrin, and monopalmitin were very low, their HPLC peaks were not identified and thus were not used as reference peaks for similarity evaluations. Ultimately, ten batches of QFY recipe were compared and analyzed (Supplementary Table S1) and proportions of constituent compounds within the ten batches ranged in similarity from 95 to 98%. The abovementioned results indicated that QFY retained the principal active components of its four constituent Chinese herbs and that different batches of the formulation exhibited good repeatability (Figure 1D).

**TABLE 1:** In the compound discoverer 3.1 library for the analysis of 19 compounds using HPLC-MS/MS.

No	RT (min)	Identification	Molecular formula	Expected neutral Mass(Da)	Observed neutral Mass(Da)	LC/MS(ESI−) (*m/z*)	Mass accuracy (ppm)		Adducts
1	1.189	Cytidine	C_9_ H_13_ N_3_ O_5_	243.08544	243.08552	244.0926	−0.33	[M+H]^+^	
2	1.588	Adenosine	C_10_ H_13_ N_5_ O_4_	267.09645	267.09675	268.10279	−1.12	[M+H]^+^	
3	6.113	Caffeic acid	C_9_ H_8_ O_4_	180.04215	180.04226	181.04947	−0.61	[M+H]^+^	
3	5.792	Caffeic acid	C_9_ H_8_ O_4_	180.04214	180.04226	179.03421	−0.62	[M−H]^−^	
4	6.345	Forsythoside B	C_34_ H_44_ O_19_	756.24826	756.24768	755.24115	0.77	[M−H]^−^	
5	6.522	Rutin	C_27_ H_30_ O_16_	610.15381	610.15338	609.14642	0.70	[M−H]^+^	
6	6.82	Forsythoside A	C_29_ H_36_ O_15_	624.20518	624.20542	625.21252	−0.38	[M+H]^+^	
7	6.983	(-)-pinoresinol 4-O-beta-D-glucopyranoside	C_26_ H_32_ O_11_	520.19442	520.19446	519.18713	−0.08	[M−H]^−^	
8	7.058	Hyperoside	C_21_ H_20_ O_12_	464.09513	464.09548	465.10214	−0.75	[M+H]^+^	
9	7.661	Iridin	C_24_ H_26_ O_13_	522.13725	522.13734	523.14453	−0.17	[M+H]^+^	
10	7.723	Phillyrin	C_27_ H_34_ O_11_	534.2086	534.210112	535.19586	−2.83	[M+H]^+^	
11	8.093	Baicalin	C_21_ H_18_ O_11_	446.08431	446.08491	447.09115	−1.34	[M+H]^+^	
12	8.729	Tectoridin	C_22_ H_22_ O_11_	462.11591	462.116212	463.12323	−0.65	[M+H]^+^	
13	8.986	Oroxindin	C_22_ H_20_ O_11_	460.10008	460.10056	461.10733	−1.04	[M+H]^+^	
14	10.118	Baicalein	C_15_ H_10_ O_5_	270.0528	270.05282	269.04553	−0.07	[M−H]^−^	
15	10.03	Tectorigenin	C_16_ H_12_ O_6_	300.06326	300.06339	299.05588	−0.43	[M−H]^−^	
15	10.341	Tectorigenin	C_16_ H_12_ O_6_	300.06308	300.06339	301.01037	−1.03	[M+H]^+^	
16	11.197	Chrysin	C_15_ H_10_ O_4_	254.05766	254.05791	253.05038	−0.98	[M−H]^−^	
17	11.283	Irisflorentin	C_20_ H_18_ O_8_	386.09958	386.100168	387.10693	−1.52	[M+H]^+^	
18	11.384	Wogonin	C_16_ H_12_ O_5_	284.06839	284.06847	283.0611	−0.28	[M−H]^−^	
19	17.467	monopalmitin	C_19_ H_38_ O_4_	330.27645	330.27701	331.28357	−1.69	[M+H]^+^	

### Impact of QFY on Lung Pathology and Inflammatory Cytokines in *S.pn*-Induced Pneumonia Mouse Model

Mouse lung tissues on slides were prepared after 48 h of infection with *S.pn* (2.5 × 10^8^ CFU/ml). Histological changes in lung tissues of mice were examined to evaluate the protective effect of QFY against the development of a pulmonary inflammatory response *in vivo*.

**FIGURE 2:**
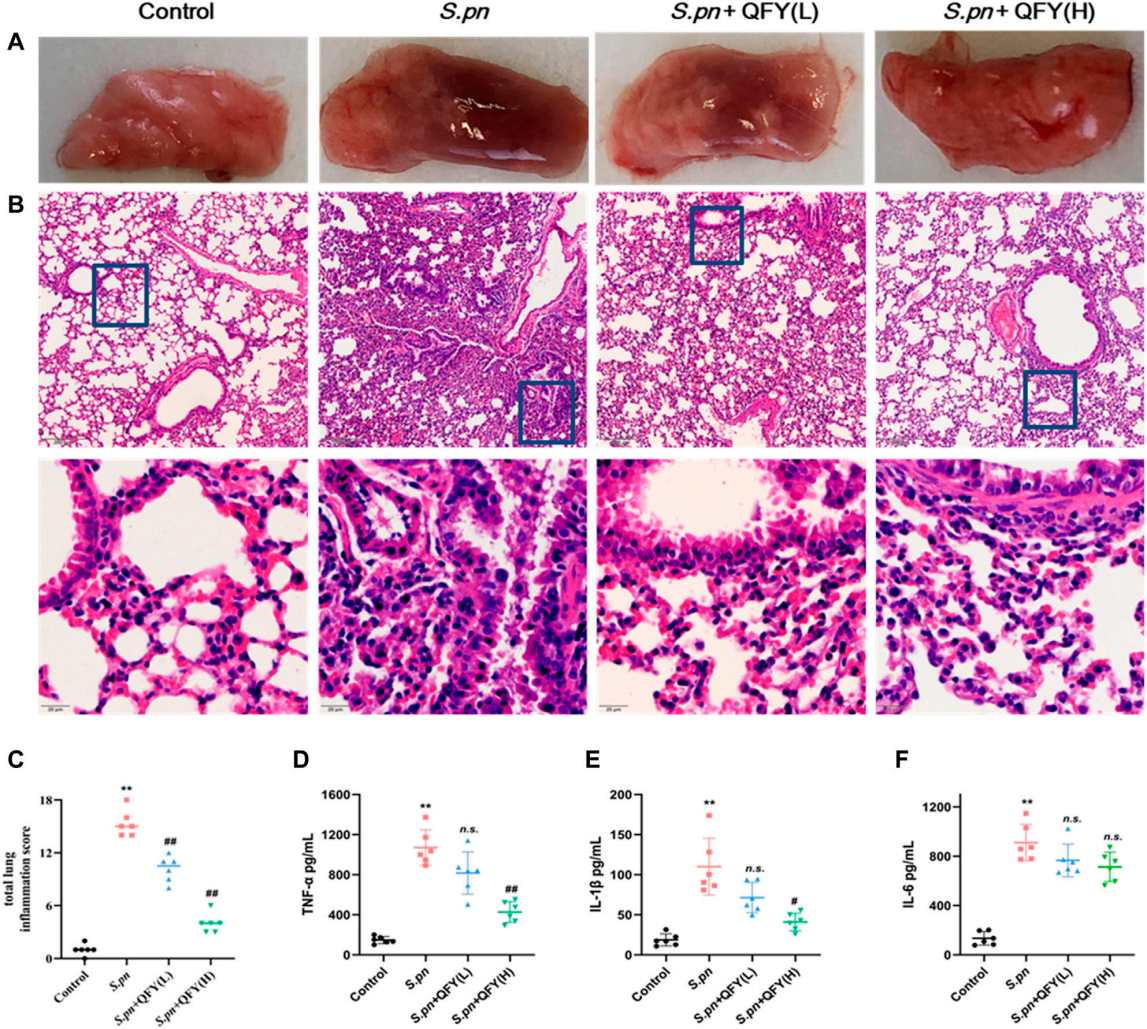
QFY prevented *S.pn*-induced lung inflammatory injury in infected mice. *S.pn*, *S.pn +* QFY(L), and *S.pn* + QFY(H) groups of mice were inoculated with 2.5 × 10^8^ CFU/ml *S.pn* in 25 μl 48 h post-infection. **(A**,**B)** Pathologic and histopathological changes in lung tissues. Lung tissues stained with H&E (original magnification ×40, scale bar: 20 μm). **(C)** Total lung inflammation score. **(D–F)** Influence of QFY on inflammatory factors detected in infected mice. Data are presented as means ± SD of *n* = 6 mice per treatment group. *p < 0.05, **p < 0.01 versus Control, ^#^ *p* < 0.05 ^##^ *p* < 0.01 versus *S.pn*, with n.s. used to indicate results were not significant.

Representative lung histology of mice showed extensive signs of pneumonia, with deepened color of lung tissue observed that was accompanied by interstitial inflammation, vasculitis, bronchitis, and edema. Lobes were congested and swollen and exhibited evidence of neutrophil infiltration. After QFY treatment, lung tissue bleeding and swelling were reduced. Based on the semi-quantitative scoring system described in the Methods section, histopathology scores were much lower in QFY-treated mice, especially for the *S.pn* + QFY (H) group, indicating that QFY treatment of mice prevented lung inflammatory damage induced by *S.pn* (Figures 2A–C).

We next measured levels of inflammatory cytokines in BALF to investigate effects of *S.pn* infection on lung inflammation and protective effects of QFY against *S.pn*-induced pneumonia. As shown in Figures 2D–F, *S.pn* infection led to significantly increased inflammatory cytokine levels of TNF-α, IL-1β, and IL-6 in BALF (*p* < 0.01). In addition, QFY supplementation significantly decreased levels of IL-1β and TNF-α as compared to corresponding levels in *S.pn*-infected mice, while IL-6 levels were not significantly decreased (*p* > 0.05). Thus, the abovementioned data revealed that *S.pn* infection induced lung inflammation that was reversed by QFY treatment. Here we note that for the *S.pn+*QFY group, descriptions of proteomics and western blotting analysis results refer to results of high-dose QFY (0.42 g/kg) experiments unless otherwise indicated. Notably, no significant differences in bacterial loads in lungs of mice were observed after QFY administration (Supplementary Figure S1).

### QFY Treatment Alters Protein Expression in *S.pn*-Infected Mice

**FIGURE 3:**
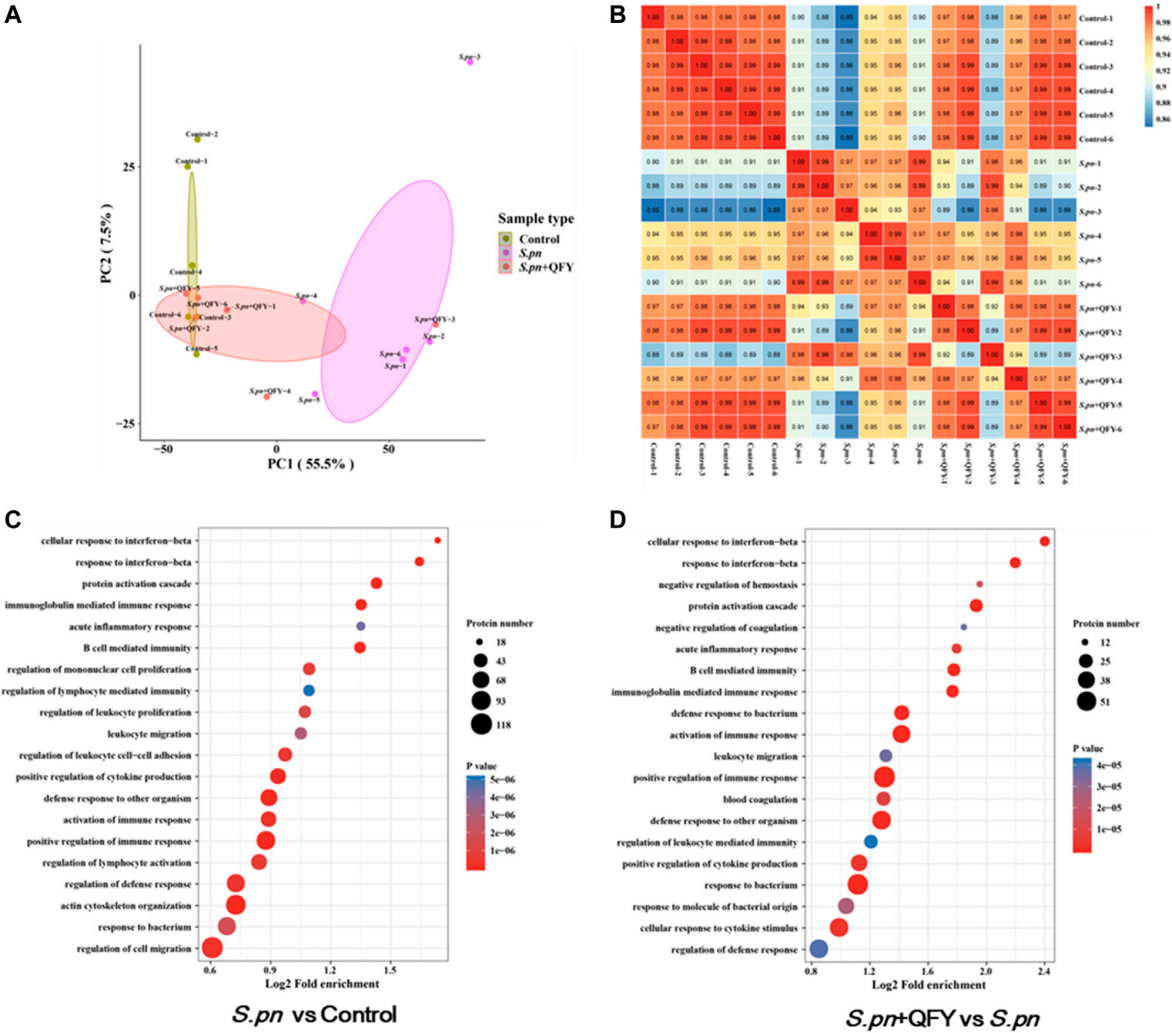
Group classification and GO functional annotation. **(A)** Overall visualization of group classifications using a PCA score plot. **(B)** Pearson correlation coefficient analysis was used to detect linear correlations between two groups of data. When the Pearson coefficient (r) was closest to −1, data groups were negatively correlated; if r was closest to 1, then data groups were positively correlated; if r was closest to 0, no correlation between data groups was found. **(C**,**D)** GO enrichment analyses of differentially expressed proteins were shown in a senior bubble chart. Bubble size indicates the number of proteins belonging to a given pathway and the color represents the *p*-value.

In order to reveal precise molecular mechanisms whereby QFY alleviated *S.pn*-induced pneumonia in mice, 4D label-free quantitative proteomics analysis was used to examine differentially expressed proteins in lung tissues of QFY-treated *S.pn*-infected mice. Here, repeatability of protein quantitation was evaluated using principal component analysis (PCA) based on Pearson’s correlation coefficients. A PCA score plot revealed overall classifications among groups (Figure 3A). The tendency of groups to cluster between control and *S.pn*-infected groups was apparent, with the QFY-treated group (0.42 g/kg) cluster shifting toward the Control group (even though two control samples were misclassified as belonging to *S.pn*-infected group), indicating QFY treatment was effective. Pearson correlation coefficients of quantified expression values (after a log_2_ transformation) were calculated for all samples (Figure 3B) and led to the strengthening of the linear correlation between Control and QFY-treated group results (r > 0.97, with the exception of the *S.pn* + QFY-3 sample).

Of the total 5795 proteins that were quantified, 655 differential proteins were identified in the *S.pn* group versus the Control group (2-fold change cutoff and value of *p* < 0.05), among which 433 proteins were up-regulated and 222 proteins were down-regulated. To further explore biological significance of differentially expressed proteins, proteins were categorized according to GO functional annotation terms (Figures 3C,D) and were mainly assigned to several biological processes, including regulation of immune and cytokines production, response to bacterium, and defense response. Additionally, 345 proteins were differentially expressed in the QFY-treated group as compared to the *S.pn*-infected group. As expected, these differentially expressed proteins were significantly enriched during pathogen infection relative to other processes mentioned above. Taken together, these data suggest that QFY significantly reversed *S.pn*-induced alterations of host defenses and immune response.

### QFY Treatment Alleviates *S.pn*-Induced Pneumonia *via* NOD-like Receptor and Autophagy Signaling Pathways

**FIGURE 4:**
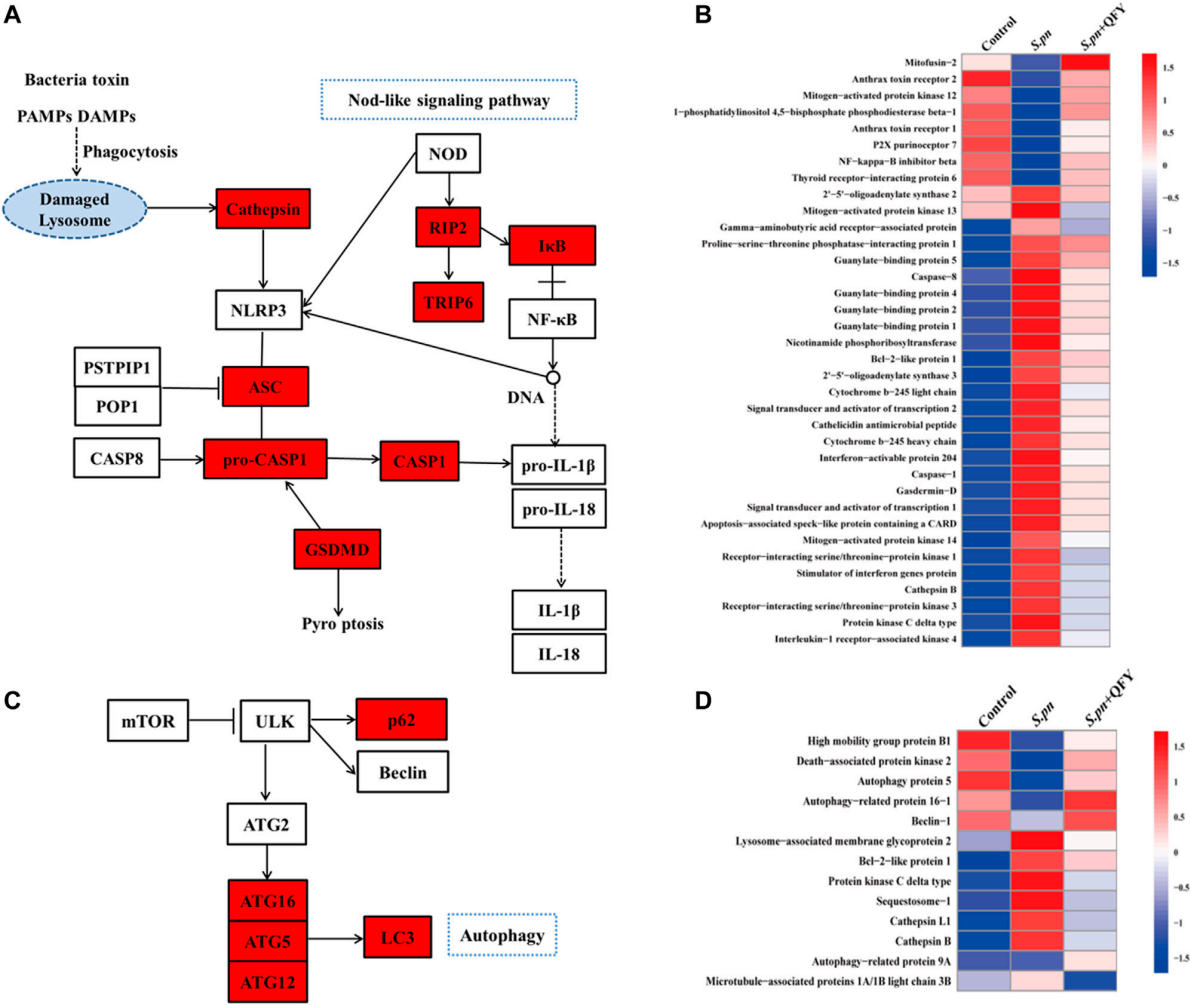
QFY alters expression of NOD-like receptor and autophagy signaling pathways. **(A**,**C)** KEGG interaction network diagram of NOD-like receptor and autophagy signaling pathway (red indicates differentially expressed proteins). **(B**,**D)** Heat maps of differentially expressed proteins of NOD-like receptor and autophagy signaling pathways (*n* = 6). Proteins were extracted, separated, and identified using LC-MS/MS analysis.

**FIGURE 5:**
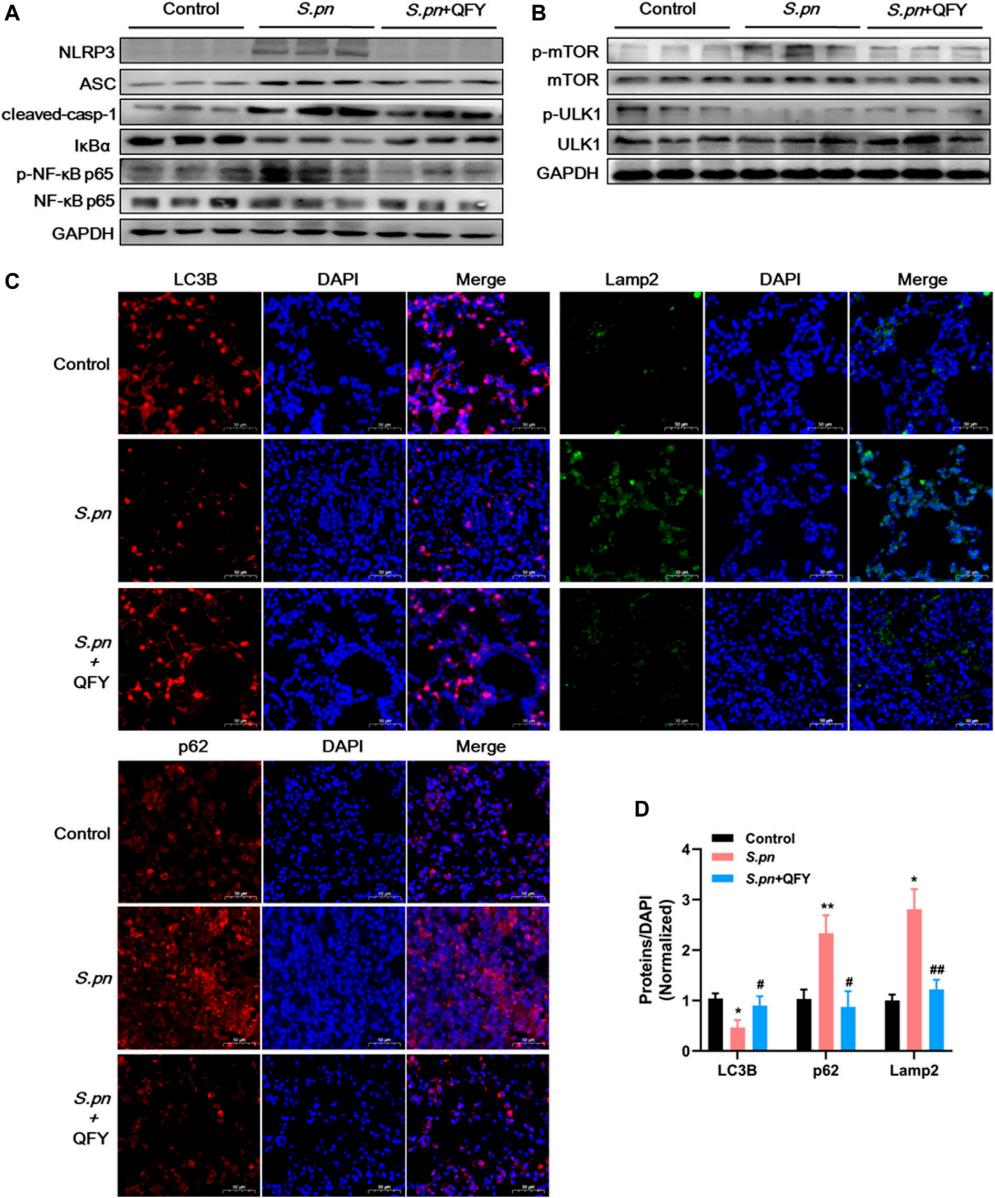
Validation of expression of key proteins associated with NLRP3 component, autophagy pathways using Western blot analysis **(A**,**B)**, and IHC **(C**,**D)** in mouse lung tissues of Control, *S.pn*, and *S.pn*+QFY groups (0.42 g/kg). Staining intensities of LC3B, p62, and Lamp2 proteins were calculated relative to DAPI staining results obtained for at least six areas of interest, with results pooled from four independent experiments. Statistical analysis was performed using one-way ANOVA followed by Tukey’s post-test, with data presented as the mean ± SD for each group, *p < 0.05, **p < 0.01 versus Control, ^#^ *p* < 0.05 ^##^ *p* < 0.01 versus *S.pn*.

To further investigate the QFY target and QFY mechanism of action during *S.pn* infection, a KEGG-based biological analysis of differentially expressed proteins was performed. As shown in Figures 4A–D, 36 and 13 differential proteins were enriched in NOD-like receptor and autophagy signaling pathways, respectively. Using Western blotting and immunohistochemistry (IHC), representative differentially expressed proteins were further verified. NLRP3, a well-known NOD-like receptor, was activated by *S.pn* invasion but was significantly down-regulated after subsequent QFY treatment. Notably, NLRP3 and NF-κB signaling pathways jointly participated in cytokines release, thus verifying involvement of other representative proteins in the abovementioned signaling pathways. As shown in Figure 5A, levels of ASC, cleaved-casp1, and phosphorylated NF-κB p65 proteins were increased after *S.pn* infection, with QFY treatment markedly restoring expression of these proteins to normal levels.

Notably, autophagy has emerged as an essential process for maintaining host homeostasis during pathogen invasion (Wu et al., 2020) (Liu et al., 2018). As shown in Figure 5B, mTOR-dependent autophagy was inhibited, as evidenced by mTOR phosphorylation and p62 accumulation at 48 h post-*S.pn* infection (Figures 5C,D). Moreover, altered autophagy observed after *S.pn* infection was further supported by observations of decreased LC3B and increased Lamp2 fluorescence intensities (Figures 5C,D). Conversely, QFY treatment enhanced host defense capacity by promoting autophagy, as indicated by QFY blockage of mTOR phosphorylation and restoration of LC3B levels that had decreased after *S.pn* infection. Corroboration of the abovementioned data indicated that QFY alleviated *S.pn*-induced pneumonia by down-regulating the level of NLRP3 and by correcting defective autophagy. We speculate that QFY may exert a similar protective effect in *in vivo* even in the absence of direct exposure to intact *S.pn* organisms, prompting us to test this hypothesis by studying PLY-treated MH-S cells as an *in vitro* pneumonia model.

### Effect of QFY on PLY-Induced MH-S Cells

**FIGURE 6:**
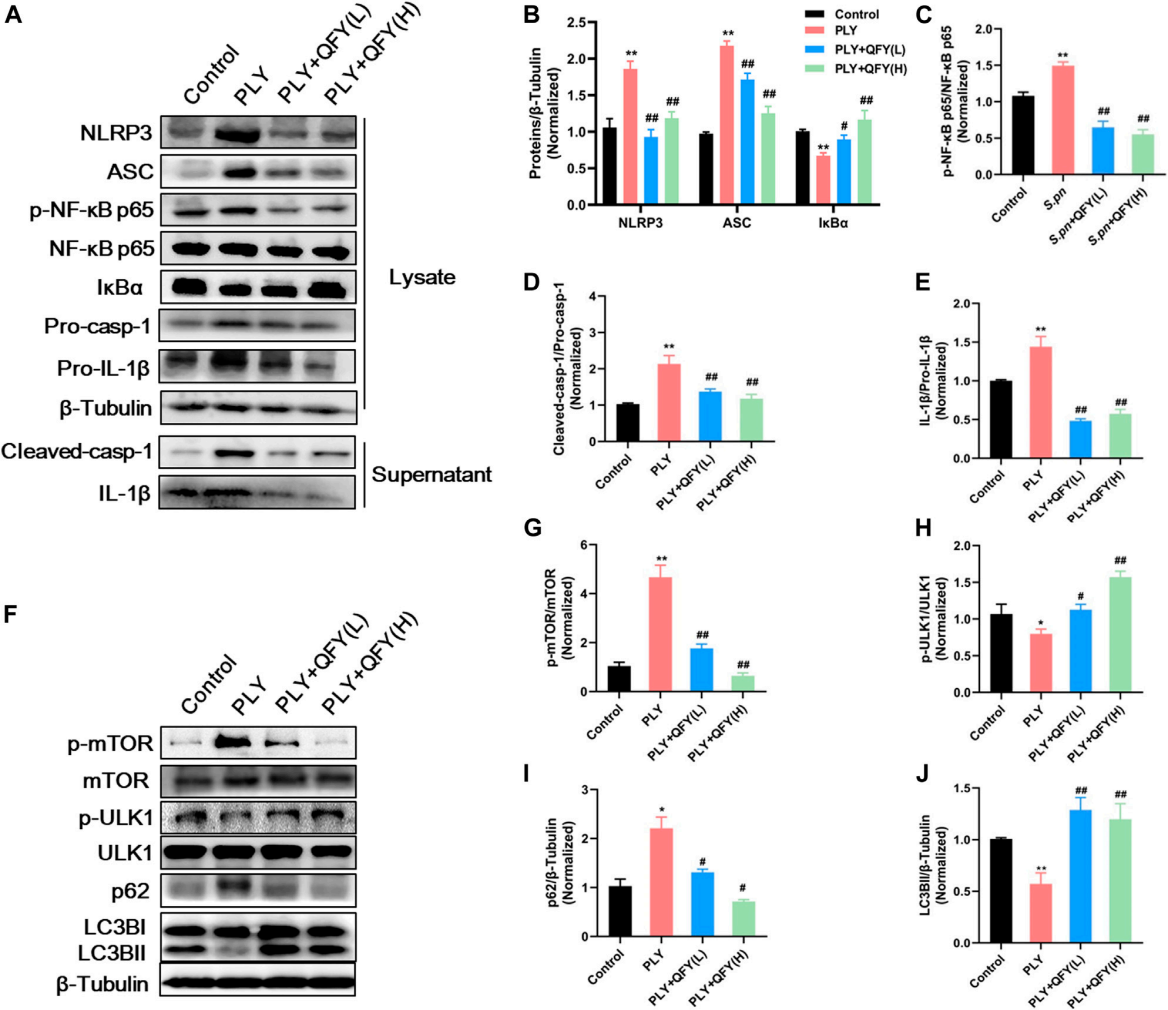
**(A)** and **(F)** protein expression detected by Western blotting. Proteins were quantified in **(B–E)** and **(G–J)**, data are presented as the mean ± SD for each group (*n* = 3), ^∗^ *p* < 0.05, ^∗∗^ *p* < 0.01 versus Control, ^#^ *p* < 0.05 ^##^ *p* < 0.01 versus PLY.

PLY, by triggering cell death and evading several host defense mechanisms, appears to directly increase the level of NLRP3 during streptococcal infection (Subramanian et al., 2019). To further explore whether QFY protective alleviation of *S.pn* infection was associated with NLRP3 down-regulation (and autophagy activation as well), we investigated expression levels of key proteins within PLY-treated MH-S cells. Here, MH-S cells were co-treated with PLY (400 ng/ml) and low-dose (25 μg/ml) or high-dose (50 μg/ml) QFY for 24 h (Figure 6). In agreement with *in vivo* experimental results obtained here, PLY induced increases in levels of NLRP3, ASC, and cleaved-casp1 proteins, with dysfunctional autophagy also detected, as revealed by increases of mTOR phosphorylation and p62 accumulation. In addition, the NF-κB signaling pathway was activated, as confirmed by the observed reduction of IκBα (decreased by 31.33% compared with the Control group) and by phosphorylation of NF-κB p65 (increased by 66.7% compared with the Control group) that together intensified the inflammatory cascade. Taken together, the abovementioned results indicate that the QFY therapeutic effect is linked to reduction of NLRP3 and activation of autophagy.

Importantly, it has been reported that NLRP3 and autophagy pathways are linked by reciprocal regulation (Zhou et al., 2011; Salminen et al., 2012; Shi et al., 2012); mechanistically, autophagy is essential for NLRP3 degradation, while NLRP3 can act as a novel inhibitor of autophagy (Cosin-Roger et al., 2017). These observations inspired us to further explore bidirectional regulatory mechanisms involving NLRP3 and autophagy pathways and to identify QFY targets.

### QFY Activates Autophagy by Down-Regulating Upstream NLRP3/mTOR Pathways

**FIGURE 7:**
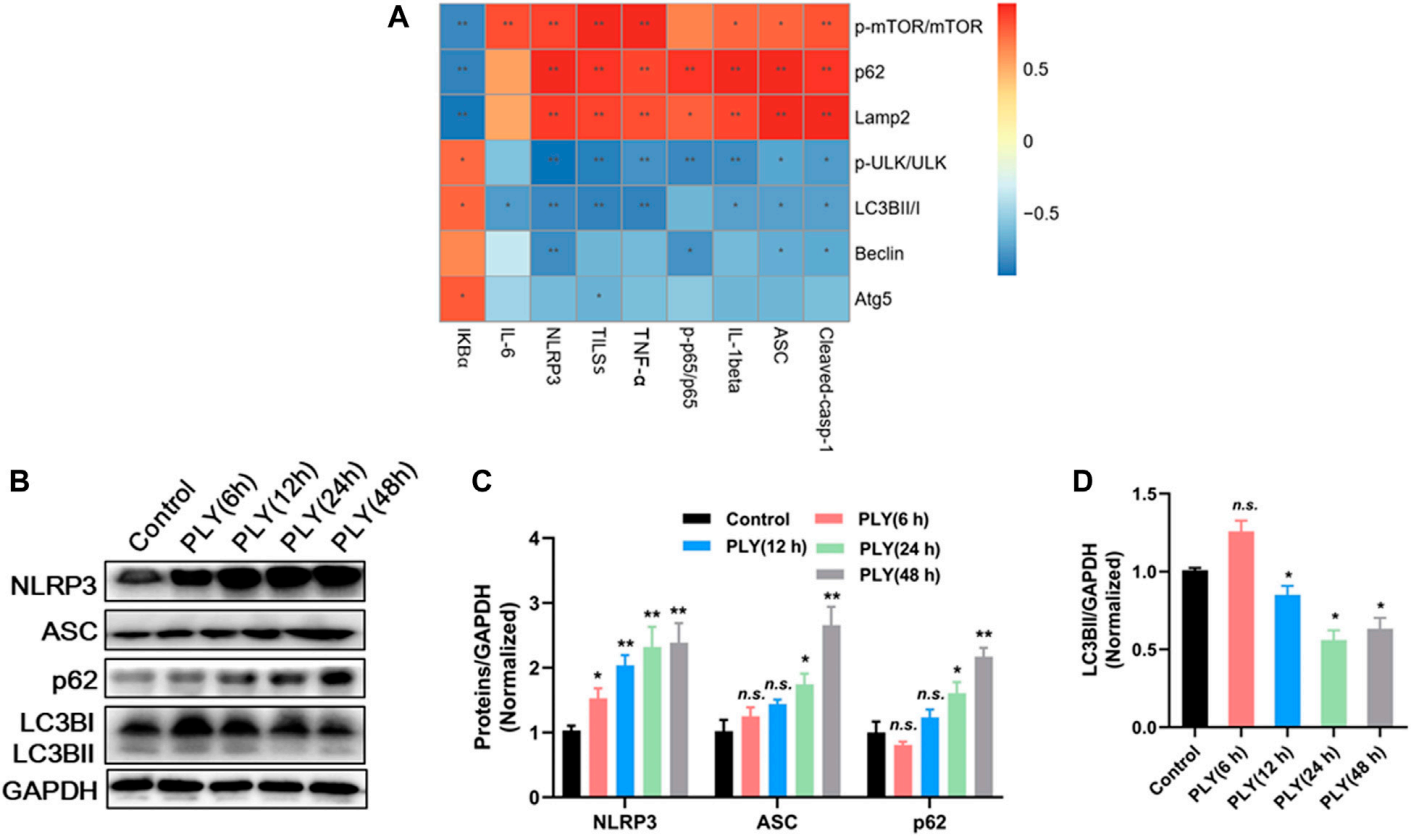
**(A)** Spearman correlation analysis of autophagy and NLRP3 signaling pathways. **(B)** The response of MH-S cells against PLY stimulation (400 ng/ml) was explored *via* a time-course experiment conducted from 6 to 48 h post-stimulation. Proteins were quantified in **(C**,**D)**, with data are presented as mean ± SD for each group (*n* = 3), *p < 0.05, **p < 0.01 versus Control.

Mounting evidence has revealed that up-regulation of NLRP3 expression depends on activation of the NF-κB pathway. Meanwhile, NLRP3 acts as a binding partner of mTOR that engages in formation of NLRP3-mTOR complexes that inhibit autophagy (Cosin-Roger et al., 2017) and thus inhibit NLRP3 degradation, an autophagy-dependent process (Shi et al., 2012). Based on these observations, here Spearman correlation analysis was performed to assess the potential association between levels of NLRP3 pathway proteins and autophagy. Intriguingly, a highly significant positive correlation was observed between autophagy-related protein tool lung inflammatory scores (TLISs) and levels of inflammatory cytokines IL-1β and TNF-α. Conversely, NLRP3, ASC, and caspase-1 levels were significantly positively correlated with p-mTOR/mTOR and p62 levels and significantly negatively correlated with LC3BII/I and p-ULK/ULK levels (Figure 7A). Taken together, these findings indicate that development of pneumonia is accompanied by blockage of autophagy, with NLRP3 and autophagy pathways negatively associated with one another.

Next, the response of MH-S cells against PLY stimulation (400 ng/ml) was explored *via* time-course experiments conducted from 6 to 48 h. As shown in Figures 7B–D, protein expression levels of NLRP3 and ASC were not significantly altered at 6 h post-infection. However, after prolonged infection for 48 h, expression of NLRP3 and ASC increased more than 3-fold as compared with their respective basal levels. As expected, PLY treatment induced transiently increased autophagy after 6 h as a defense response; this response was significantly reduced as NLRP3 continued to accumulate, as evidenced by a decrease of LC3BII/GAPDH by 58.56% as compared with the Control group. Since our abovementioned results suggest a relationship between NLRP3 and autophagy, we sought to elucidate the exact molecular target of QFY using NLRP3 siRNA and the PI3K inhibitor 3-methyladenine (3-MA).

**FIGURE 8:**
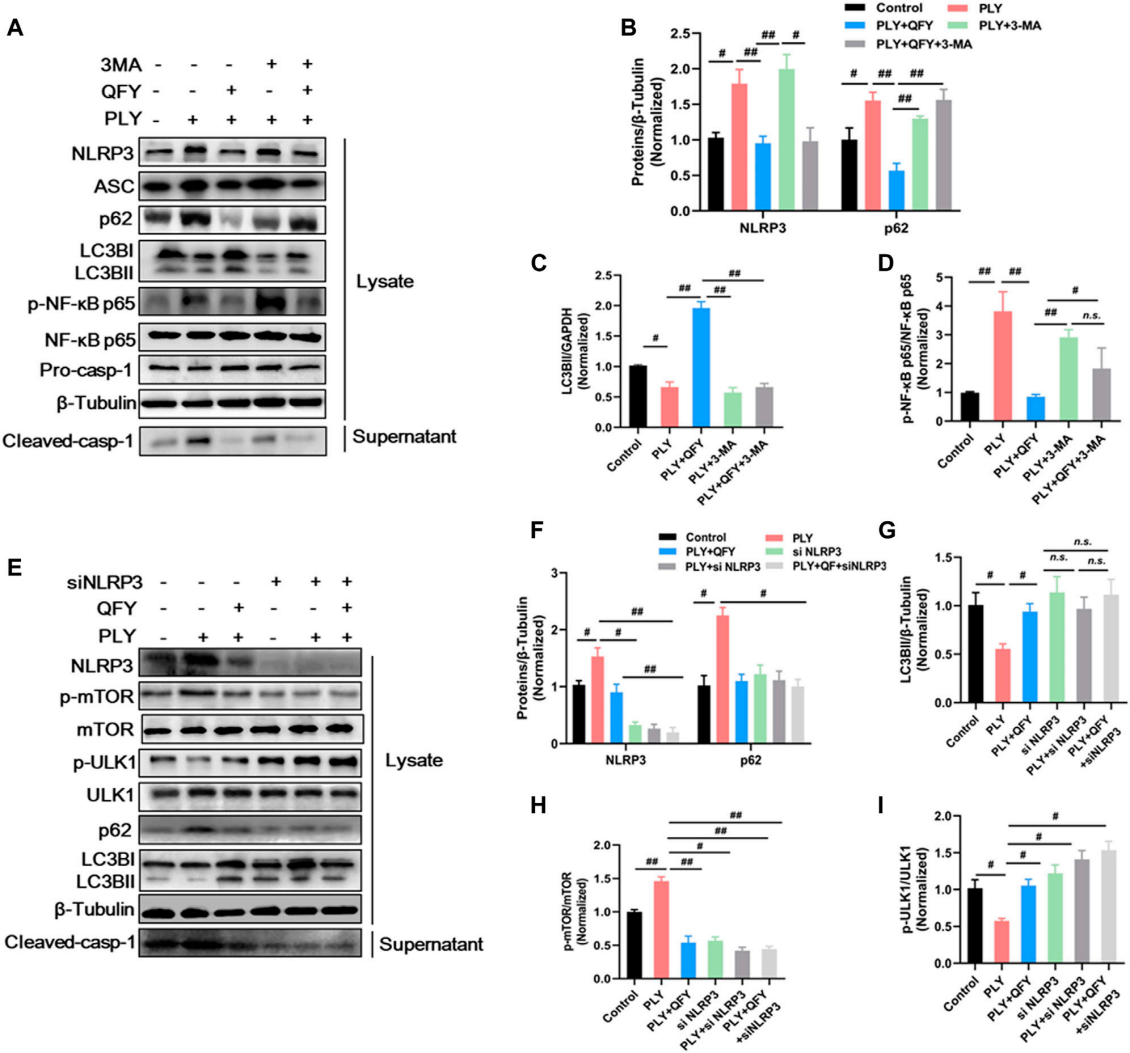
QFY activates autophagy by down-regulating the upstream NLRP3/mTOR pathway. MH-S cells were stimulated with purified PLY (400 ng/ml) to induce the lung injury model then were treated with QFY (50 μg/ml) for 24 h **(A**,**E)** protein expression detected by Western blotting. Proteins were quantified in **(B**–**D)** and **(F**–**I)**, data are presented as the mean ± SD for each group (*n* = 3), ^#^ *p* < 0.05, ^##^ *p* < 0.01.

Concurrent with our abovementioned data, QFY treatment significantly down-regulated NLRP3 pathway component and also repaired defective autophagy (Figure 8A, lanes 1–3), while 3-MA inhibited the autophagy response, as indicated by increased p62 and decreased LC3B levels (as shown in Figure 8A, lanes 4–5, respectively). Notably, treatment with QFY reversed PLY-induced increases in NLRP3 pathway member levels even in the presence of 3-MA. Thus, these results suggest that QFY suppressed the PLY-induced inflammatory response by inhibiting NLRP3 rather than by triggering the autophagy pathway. Next, we further explored whether NLRP3 is the target of QFY modulation of autophagy and amelioration of inflammation. As shown in Figure 8E, lane 4, silencing of NLRP3 expression markedly restored the basal autophagy level, indicating that NLRP3 degradation may be involved in activation of autophagy (Cosin-Roger et al., 2017).

In summary, the QFY anti-streptococcus pneumonia effect appears to be associated with activation of autophagy through down-regulation of upstream autophagy pathway events involving NLRP3/mTOR.

## Discussion

Major conclusions of this study can be summarized as three points. First, QFY exerts prominent anti-pneumonia effects both in a *S.pn*-induced *in vivo* mouse model and an *in vitro* PLY-stimulated MH-S cell-based model. Second, 4D label-free proteomics analysis showed that suppression of NLRP3 expression and repair of autophagy played pivotal pharmacological roles in QFY pneumonia therapy. Third, our data and previously reported results of other studies demonstrated that NLRP3 and autophagy were closely linked. Here we found that QFY triggered autophagy *via* inhibition of upstream NLRP3/mTOR signaling pathway events, resulting in the amelioration of inflammatory injury.

Lower respiratory tract infections, as reported in the Global Burden of Diseases, Injuries, and Risk Factors Study (GBD), caused 2.38 million deaths in 2016 and thus are a serious public health concern. Among children, those younger than 5 years of age accounted for 27.45% of these deaths. *Streptococcus pneumoniae*-induced pneumonia is the main cause of lower respiratory tract infections, which are responsible for more than 1.189 million deaths per year (D 2016 Lower Respirator, 2018).

Nowadays, antibiotic resistance has led to the declining effectiveness of traditional antimicrobial agents used to combat *S.pn* infections, necessitating the development of strategies for reducing antibiotics application (Quinton et al., 2018; Kadri and Boucher, 2020). TCM has been practiced for thousands of years and is widely recognized as therapeutically curative when used for treatment of several diseases and physiological conditions (Wang et al., 2017). In fact, treatment efficacies of some TCM-based approaches have been experimentally confirmed (Qiu et al., 2012; Lai et al., 2014; Chen et al., 2015; Peng et al., 2019). According to the theory of TCM, “heat toxin” is closely related to pneumonia. QFY exerts effects that remove dampness and heat and detoxify tissues and thus is efficacious when used to treat pneumonia. Among QFY ingredients, baicalin (Qiu et al., 2012; Zhang et al., 2020), phillyrin (Zhong et al., 2013), and Forsythia suspensa (Thunb.) Vahl, possess antibacterial and anti-inflammatory activities, while Scutellaria baicalensis Georgi radix has been reported to inhibit cancer cell growth and attenuate inflammation (Ma et al., 2013; Gong et al., 2020).

Nevertheless, due to the variability and complexity of TCM formulations made from Chinese herbal compounds, their efficacies have been frequently questioned due to the lack of rigorous scientific evidence supporting their effectiveness. Thus, studies of TCM herbal compound QFY that align with biomedical perspectives are pivotal to its acceptance in clinical settings. Therefore, we established *S.pn*-infected mouse-based and PLY-stimulated MH-S cell-based lung infection models to investigate the mechanism underlying QFY protective effects against pneumonia.

HE staining of lung tissues of mice with *S.pn*-induced pneumonia showed extensive signs of infection, such as congested and swollen lobes accompanied by neutrophil infiltration. However, QFY treatment could significantly alleviated pathological manifestations of lung infection and reduced levels of cytokines IL-1β and TNF-α, with the exact mechanism of action of QFY discussed in detail below.

High-throughput proteomics tools are widely recognized as valuable for use in exploring pharmacology of complex TCM systems (Zhang et al., 2014; Dai et al., 2018; Shen et al., 2019). Here we identified 36 and 13 differentially expressed proteins that were enriched for NOD-like receptor and autophagy signaling pathways, respectively. Autophagy is an essential homeostatic process which is triggered by danger signals, including pathogen invasion. In line with recent studies linking autophagy and infectious disease (Hu et al., 2016), our results suggest that lung inflammatory injury reflects deficient autophagy, as demonstrated by p62 accumulation and decreased LC3B levels. Conversely, several research studies have demonstrated that *S.pn* stimulates autophagy during the early phase of infection (Kim et al., 2015). We hypothesized that these results reflect differing responses to *S.pn* at different infectious disease time points, prompting us to investigate this discrepancy by conducting a time-course experiment. Subsequently, it became obvious that autophagy was slightly increased at 6 h post-infection, thus reflecting its defense response role. However, autophagy decreased with prolongation of infection duration, especially after 48 h of infection. Interestingly, reduction of autophagy was accompanied by constitutive NLRP3 accumulation.

In lung tissues during early pneumococcal pneumonia, NLRP3 appears to play an increasingly important role in protective immunity as infection duration and bacterial burden increase. For instance, it has been reported that both Nlrp3^−/−^ and Asc^−/−^ mice exhibited strongly improved host defenses (as compared to mice with functional alleles for each gene) and had markedly reduced mortality rates and diminished bacterial growth and dissemination (McNeela et al., 2010b). Importantly, recognition of pneumococcal peptidoglycan and DNA by the NOD-like pathway depends on expression of PLY, which is a well-known and important *S.pn* virulence factor. PLY acts by creating transmembrane pores in membranes of pulmonary epithelial cells and macrophages, resulting in cell lysis and necrosis (Malley et al., 2003). Consequently, the immune system employs various distinct types of NOD-like receptors to sense PLY-induced cellular membrane damage (Koppe et al., 2012).

Collectively, results obtained in this study suggested that *S.pn* infection up-regulated the level of NLRP3 and markedly inhibited autophagy. By contrast, QFY-treatment significantly inhibited the expression of the NLRP3 signaling pathway, while correcting defective autophagy through mTOR dephosphorylation and p62 degradation. Unexpectedly, the abovementioned data revealed mechanistic parallels between signaling pathways leading to decreased NLRP3 levels and activation of autophagy that were observed during QFY treatment. Indeed, our results may align with results of several previous studies that suggested that NLRP3 is a binding partner of mTOR, whereby binding of NLRP3 to mTOR inhibited autophagy by promoting mTOR phosphorylation (Zhou et al., 2011; Shi et al., 2012). Interestingly, it has been recognized that NF-κB p65 is a transcription factor associated with expression of NLRP3 and cytokines, as supported by results of this work, such that changes in levels of NLRP3 mRNA and pro-IL-1β exhibited the same trends as observed for phosphorylated NF-κB p65.

**FIGURE 9:**
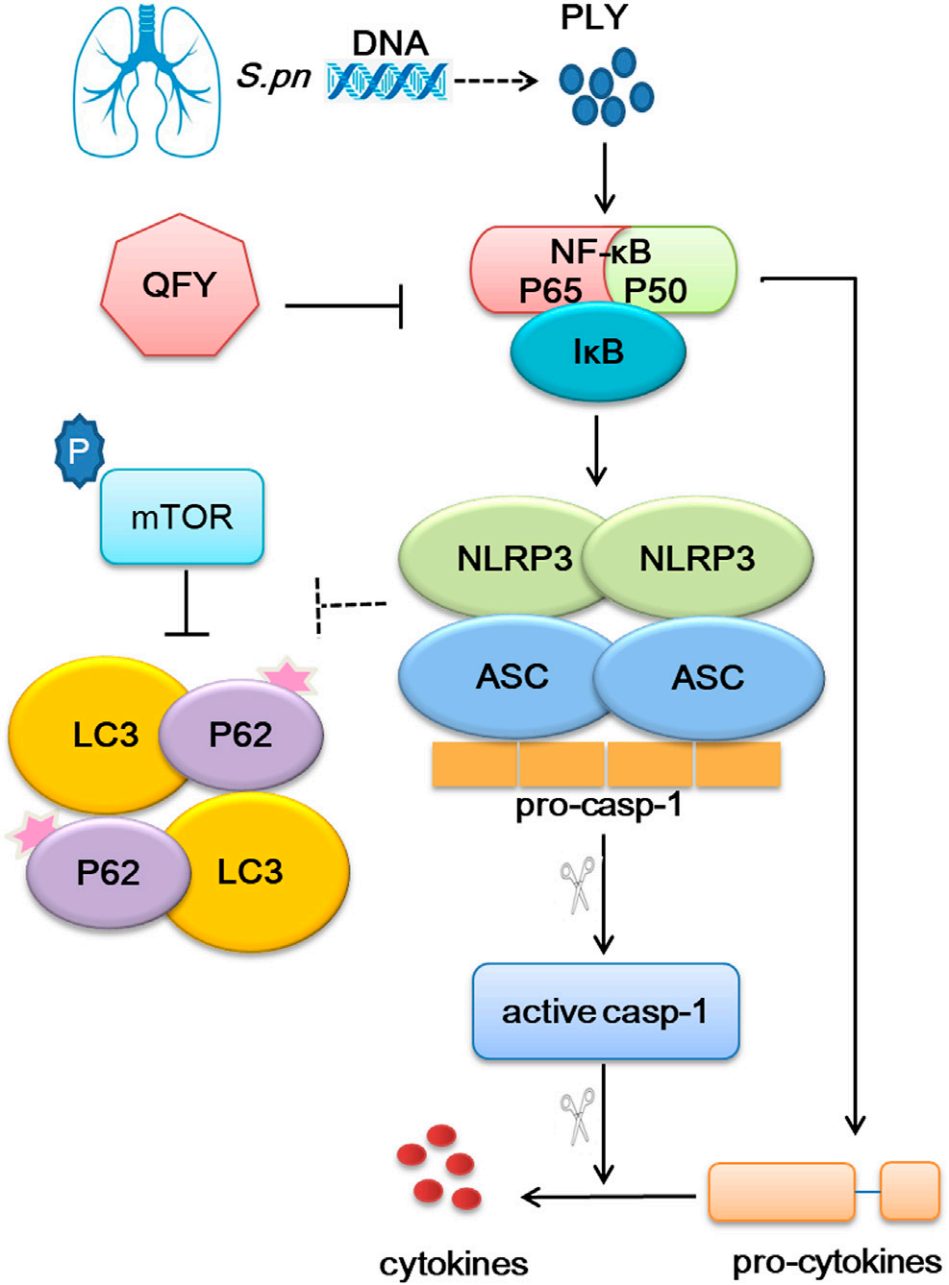
Overview of QFY therapeutic mechanism for alleviating pneumonia. QFY triggered autophagy *via* down-regulation of upstream NLRP3/mTOR signaling pathway events, resulting in of the amelioration of inflammatory injury.

To further explore precise targets involved in QFY regulation of NLRP3 and autophagy, 3-MA served as a specific autophagy inhibitor, while siRNA was used to silence NLRP3 expression. Our results confirmed that QFY treatment reversed PLY-induced NLRP3 increase even in the presence of 3-MA, while the NLRP3 siRNA mimicked QFY action by triggering mTOR-dependent autophagy. Notably, mTOR, a highly conserved serine/threonine protein kinase consisting of two mTOR complex forms, seemed to be the biochemical link between NLRP3 and autophagy signaling pathways, as shown in Figure 9.

In summary, our results demonstrate that NLRP3 inhibited autophagy by binding to mTOR, while QFY reversed infection-impaired autophagy through down-regulation of NLRP3. This study provides a rationale warranting future investigations into precise targets and mechanisms of QFY and other Chinese herbal remedies for treating lung infectious diseases.

## Data Availability

The mass spectrometry proteomics data have been deposited to the ProteomeXchange Consortium via the PRIDE (1) partner repository with the dataset identifier PXD029654.
