# Supplementary material for: Activation of Autophagy Through the NLRP3/mTOR Pathway: A Potential Mechanism for Alleviation of Pneumonia by QingFei Yin
Source: Front Pharmacol. 2022 Jan 17;12:763160. doi: 10.3389/fphar.2021.763160 (PMC8802069; doi:10.3389/fphar.2021.763160)
Supplement: Supplementary file 1 [file DataSheet1.docx]

Supplementary Material

Activation of autophagy through the NLRP3/mTOR pathway: a potential mechanism for alleviation of pneumonia by QingFei Yin

**Xiaozhou Sun^1^**^†^**, Dandan Wang^1, 2^**^†^ **, Lizhong Ding^1, 3^ Yan Xu^1^, Wenxiu Qi^4^ , Daqing Zhao^4^, Li Liu^5^, Chengcheng Yin^5^ , Changsheng Cui^5^ , Zhongtian Wang^1^ , Liwei Sun^1, 2*^, Liping Sun^1, 3*^**

^1^ College of Chinese Medicine, Changchun University of Chinese Medicine; Changchun, Jilin, China

^2^ Research Center of Traditional Chinese Medicine, the Affiliated Hospital to Changchun University of Chinese Medicine, Changchun, Jilin, China

^3^ Center of Children's Clinic, the Affiliated Hospital to Changchun University of Chinese Medicine, Changchun, Jilin, China

^4^ Jilin Provincial Key Laboratory of Bio Macromolecules of Chinese Medicine, Jilin Ginseng Academy, Changchun University of Chinese Medicine, Changchun, Jilin, China

^5^ College of Pharmacy, Changchun University of Chinese Medicine, Changchun, China

^†^ Co-first authors

*** Correspondence:**

Corresponding Author

E-mail addresses: sunnylilwei@163.com (L. Sun).

E-mail addresses: [slpcczyydx@sina.com](mailto:slpcczyydx@sina.com) (L. Sun).

**1. Methods and material**

**1.1 Measurement of Bacterial Loads.**

The bacterial numbers were identified by colony counts of lung tissue smears as previously described [1; 2]. Lungs were collected from euthanized mice, and lung tissue homogenates were prepared in 1 ml of sterile PBS at 4°C and used to calculate the bacterial colony counts through the serial dilution method and smearing on solid media.

**1.2 Quantitative real-time (qRT-PCR)**

The concentration of D39 DNA in murine serum in mouse lung tissue was measured by qRT-PCR. Briefly, total RNA was isolated using the TriPure™ reagent (Roche, Germany, 11667165001) according to the manufacturer’s instructions. For determination of mRNAs expression, cDNA was synthesized using 1μg of total RNA reverse transcription-primers (Roche, Germany, 4897030001). The primers used for real-time PCR targeted a fragment of the pneumolysin-encoding gene of *S. pneumoniae* (Forward: 5′-AGCGATAGCTTTCTCCAAGTGG-3′,Reverse:5′-CTTAGCCAACAAATCGTTTACCG-3′) [2] as the normalization control. qRT-PCR reactions were done with FastStart ™ General SYBR Green premix (Roche, Germany, 4913914001). The relative levels of genes expression were quantified using the ΔCt-ΔCt method

**1.3 HPLC Fingerprint Analysis**

The QFY aqueous extract was placed in a conical flask then it was mixed with 10 mL 70% ethanol. After placing a cork in the flask, the mixture was shocked for 40 min (power 250 W, frequency 50 kHz). After shocking, the mixture was centrifuged at 4000 rpm/min for 15 min to yield a supernatant containing QFY that was then filtered (0.22 μm). Ten batches of QFY compound from different sources were each separated using a ZORBAX Eclipse Plus C18 (4.6 mm × 250 mm, 5-µm) HPLC system (Shimadzu, Kyoto, Japan) and a diode array detector (UltiMate 3000, DIONEX, Sunnyvale, CA, USA). Analysis of specific components in each batch yielded chemical fingerprints. The mobile phase condition was recorded as follows: acetonitrile (as1122-801, Tedia, Fairfield, OH, USA) in water (A) and 0.05% phosphoric acid aqueous solution (B). The column temperature was 30 °C and the flow rate was 1.0 mL/min that allowed a gradient to form. UV detection was conducted at 266 nm. Empower software (Shimadzu) was used to collect and analyze chromatographic data.

**Table S1 Source of 10 batches of QFY**

| **batch** | **medicinal materials** | **batch number** | **Manufacturer** |
| --- | --- | --- | --- |
| **S1** | **Belamcandae Rhizoma** | **A190019220** | **Peili (Nanning) Pharmaceutical Co., Ltd** |
|  | **Frsythia** | **A1701862** | **Peili (Nanning) Pharmaceutical Co., Ltd** |
|  | **Fritillaria Cirrhosa** | **A1801140** | **Peili (Nanning) Pharmaceutical Co., Ltd** |
|  | **Scutellaria baicalensis** | **A190116910** | **Peili (Nanning) Pharmaceutical Co., Ltd** |
| **S2** | **Belamcandae Rhizoma** | **18026374** | **Jiangyin Tianjiang Pharmaceutical Co., Ltd** |
|  | **Forsythia** | **19046344** | **Jiangyin Tianjiang Pharmaceutical Co., Ltd** |
|  | **Fritillaria Crrhosa** | **19056464** | **Jiangyin Tianjiang Pharmaceutical Co., Ltd** |
|  | **Scutellaria baicalensis** | **19046494** | **Jiangyin Tianjiang Pharmaceutical Co., Ltd** |
| **S3** | **Belamcandae Rhizoma** | **0065825524** | **Sichuan new green Pharmaceutical Technology Development Co., Ltd** |
|  | **Forsythia** | **0065825482** | **Sichuan new green Pharmaceutical Technology Development Co., Ltd** |
|  | **Fritillaria Cirrhosa** | **1812033** | **Sichuan new green Pharmaceutical Technology Development Co., Ltd** |
|  | **Scutellaria baicalensis** | **0065825521** | **Sichuan new green Pharmaceutical Technology Development Co., Ltd** |
| **S4** | **Belamcandae Rhizoma** | **A1800070** | **Nongbenfang 1** |
|  | **Forsythia** | **A1701863** | **Nongbenfang 1** |
|  | **Fritillaria Cirrhosa** | **A1801141** | **Nongbenfang 1** |
|  | **Scutellaria baicalensis** | **A190116911** | **Nongbenfang 1** |
| **S5** | **Belamcandae Rhizoma** | **19036074** | **Jiangyin Tianjiang Pharmaceutical Co., Ltd** |
|  | **Forsythia** | **19046344** | **Jiangyin Tianjiang Pharmaceutical Co., Ltd** |
|  | **Fritillaria Cirrhosa** | **19056464** | **Jiangyin Tianjiang Pharmaceutical Co., Ltd** |
|  | **Scutellaria baicalensis** | **19046494** | **Jiangyin Tianjiang Pharmaceutical Co., Ltd** |
| **S6** | **Belamcandae Rhizoma** | **0102942438** | **Sichuan new green Pharmaceutical Technology Development Co., Ltd** |
|  | **Forsythia** | **0102942435** | **Sichuan new green Pharmaceutical Technology Development Co., Ltd** |
|  | **Fritillaria Cirrhosa** | **1812033** | **Sichuan new green Pharmaceutical Technology Development Co., Ltd** |
|  | **Scutellaria baicalensis** | **0102942426** | **Sichuan new green Pharmaceutical Technology Development Co., Ltd** |
| **S7** | **Belamcandae Rhizoma** | **A1800069** | **Nongbenfang 2** |
|  | **Forsythia** | **A1701862** | **Nongbenfang 2** |
|  | **Fritillaria Cirrhosa** | **A1801140** | **Nongbenfang 2** |
|  | **Scutellaria baicalensis** | **A190116910** | **Nongbenfang 2** |
| **S8** | **Belamcandae Rhizoma** | **A190053610** | **Nongbenfang 3** |
|  | **Forsythia** | **A190053610** | **Nongbenfang 3** |
|  | **Fritillaria Cirrhosa** | **A190014310** | **Nongbenfang 3** |
|  | **Scutellaria baicalensis** | **A190053610** | **Nongbenfang 3** |
| **S9** | **Belamcandae Rhizoma** | **A190019220** | **Peili (Nanning) Pharmaceutical Co., Ltd** |
|  | **Forsythia** | **A1701832** | **Peili (Nanning) Pharmaceutical Co., Ltd** |
|  | **Fritillaria Cirrhosa** | **A1801120** | **Peili (Nanning) Pharmaceutical Co., Ltd** |
|  | **Scutellaria baicalensis** | **A190114210** | **Peili (Nanning) Pharmaceutical Co., Ltd** |
| **S10** | **Belamcandae Rhizoma** | **18074376** | **Jiangyin Tianjiang Pharmaceutical Co., Ltd** |
|  | **Forsythia** | **19046368** | **Jiangyin Tianjiang Pharmaceutical Co., Ltd** |
|  | **Fritillaria Cirrhosa** | **19056467** | **Jiangyin Tianjiang Pharmaceutical Co., Ltd** |
|  | **Scutellaria baicalensis** | **19046494** | **Jiangyin Tianjiang Pharmaceutical Co., Ltd** |

**2. Results**

**2.1 The effect of QFY on bacteria loading in *S.pn* infected mice.**

To evaluate the *S. pneumoniae* survival influenced by QFY, we determined the colony-forming units (CFU) and DNA content after administration of *S.pn* after 48 hours**.** The bacterial loading in the lungs was slightly lower in the QFY-treat high dose group than that of *S.pn* group with no significant differences (**Figures S1A, B**). Hence, we believed that inhibition of the bacteria survival is not the main pathway on which QFY exerts its therapeutic effect.

**
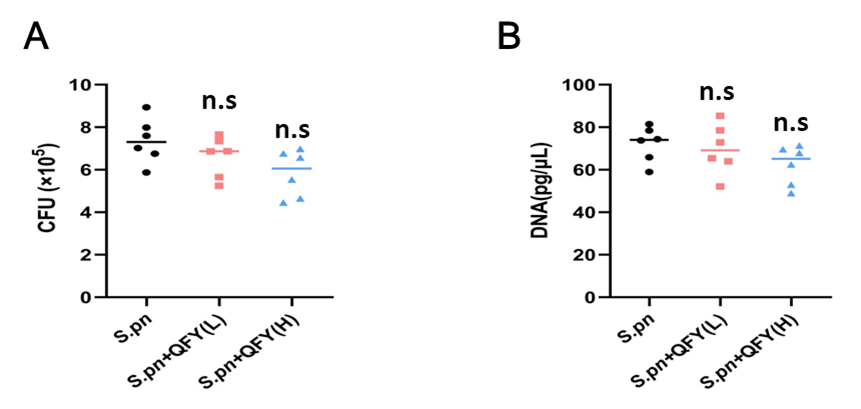
**

**Figure S1** The influence of QFY exerted on the lung bacteria burden of infected mice. **(A, B)** The infected extent could be estimated by CFU and DNA of the lung bacteria of the mice that had been influenced for 48 hours. Data are presented as means ± SD, n.s, no significant differences.

[**References**](javascript:;)

[1] X. Zhao, H. Li, J. Wang, Y. Guo, B. Liu, X. Deng, and X. Niu, Verbascoside Alleviates Pneumococcal Pneumonia by Reducing Pneumolysin Oligomers. Molecular pharmacology 89 (2016) 376-87.

[2] T. Isono, and H. Domon, Treatment of severe pneumonia by hinokitiol in a murine antimicrobial-resistant pneumococcal pneumonia model. 15 (2020) e0240329.

**
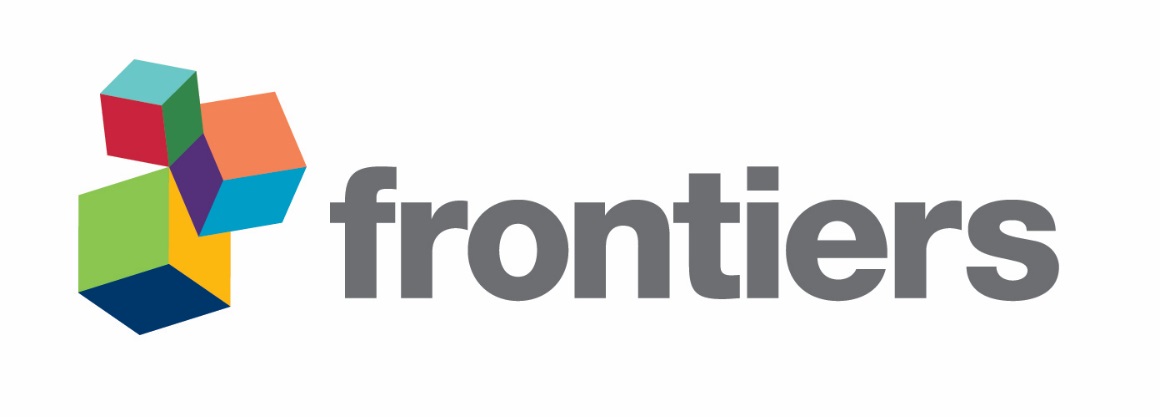
**
